# In vitro Modeling of Adipogenesis Using Mesenchymal Stem Cells from Subcutaneous Adipose Tissue to Explore the Cellular and Molecular Mechanisms Underlying Pediatric Obesity and Obesogenic Memory

**DOI:** 10.1159/ofa/adzag003

**Published:** 2026-08-14

**Authors:** Marko Dardi, Clarissa Berardo, Gloria Pelizzo, Maxime Bonnet, Francesca Destro, Gerson Capelo, Michele Ceresola, Alessia Lai, Stephana Carelli, Gianvincenzo Zuccotti, Valeria Calcaterra

**Affiliations:** a Pediatric Clinical Research Center “Romeo ed Enrica Invernizzi”, Department of Biomedical and Clinical Science, University of Milan, Milan, Italy; b Center of Functional Genomics and Rare Diseases, Buzzi Children’s Hospital, Milan, Italy; c Department of Biomedical and Clinical Sciences, University of Milan, Milan, Italy; d Pediatric Surgery Department, Children’s Hospital “Vittore Buzzi”, Milan, Italy; e Laboratory of Medical Microbiology and Virology, University of Insubria, Varese, Italy; f Department of Pediatrics, Children’s Hospital “Vittore Buzzi”, Milan, Italy; g Pediatrics and Adolescentology Unit, Department of Internal Medicine, University of Pavia, Pavia, Italy

**Keywords:** Childhood obesity, Adipogenesis, Obesogenic memory, Children, Long-term metabolic complication

## Abstract

**Introduction:**

Pediatric obesity is associated with early metabolic complications and increased risk of persistent obesity in adulthood. Beyond fat accumulation, obesity may induce long-lasting molecular alterations in adipose tissue, described as “obesogenic memory.” However, whether such intrinsic alterations are already present in pediatric adipose progenitor cells remains poorly understood. This study aimed to develop an in vitro model of adipogenesis using pediatric adipose-derived mesenchymal stem cells (hMSCs) and to investigate whether obesity is associated with intrinsic metabolic reprogramming of adipose progenitors.

**Methods:**

hMSCs were isolated from periumbilical subcutaneous adipose tissue obtained from pediatric subjects with normal weight (NW), overweight (OW), and obesity (OB). Cells were expanded and induced to undergo adipogenic differentiation for up to 14 days using defined adipogenic media. Differentiation was evaluated through morphological analysis, lipid accumulation (BODIPY staining), gene expression profiling by RT-qPCR, and protein analysis by Western blot.

**Results:**

Two differentiation protocols efficiently induced adipocyte maturation without cytotoxicity. hMSCs derived from NW and OB subjects showed comparable adipogenic differentiation capacity, with similar lipid droplet accumulation and expression of canonical adipogenic markers, including C/EBPα, PPARγ, and FABP4. Despite this comparable differentiation efficiency, OB-derived adipocytes exhibited altered transcriptional regulation of genes involved in lipid metabolism, including pathways associated with lipogenesis, lipolysis, and fatty acid β-oxidation. Protein analyses further revealed dysregulated expression of key metabolic regulators such as SCD1, SREBP1, PNPLA2, and FADS1, suggesting altered lipid-metabolic programming.

**Conclusions:**

Pediatric obesity does not impair adipogenic differentiation but is associated with intrinsic metabolic alterations in adipose progenitor cells. These findings suggest the hypothesis of the presence of obesogenic memory in adipose tissue that may contribute to long-term metabolic dysfunction.

## Introduction

Pediatric obesity represents one of the most serious and rapidly growing public health challenges worldwide. According to the World Health Organization [1], an estimated 35 million children under 5 years of age were overweight in 2024, while over 390 million children and adolescents aged 5–19 years were overweight in 2022, including 160 million living with obesity. In this age group, the global prevalence of overweight, including obesity, increased from 8% in 1990 to 20% in 2022, while the prevalence of obesity alone rose from 2% to 8%, highlighting the dramatic rise of pediatric excess adiposity over the last 4 decades [1, 2]. Obesity in childhood is no longer considered a benign or transient condition as it is strongly associated with the early development of cardiometabolic complications, including insulin resistance, type 2 diabetes, dyslipidemia, and hypertension, and it significantly increases the risk of obesity persistence into adulthood [2–7].

In recent years, obesity has increasingly been conceptualized not only as a disorder of excess fat accumulation but also as a condition characterized by profound endocrine, inflammatory, and immune alterations [8–13]. Adipose tissue is now recognized as a highly dynamic organ involved in metabolic regulation, immune signaling, and systemic homeostasis rather than a passive lipid storage depot [13–15]. In this framework, the concept of adipose tissue dysfunction has emerged as a central determinant of obesity-related complications, highlighting the importance of qualitative alterations in adipose tissue biology beyond total fat mass [14, 16, 17].

A growing body of evidence suggests that obesity induces long-lasting molecular and cellular changes in metabolic tissues that may persist even after weight normalization [18]. This phenomenon has been described as “obesogenic memory,” referring to stable metabolic, transcriptional, and epigenetic alterations acquired during exposure to an obesogenic environment [19]. Such memory may affect adipose tissue expandability, inflammatory responses, lipid metabolism, and metabolic flexibility, potentially predisposing individuals to weight regain and long-term metabolic dysfunction. Although this concept has been increasingly supported by studies in adults, direct experimental evidence in pediatric adipose tissue remains limited.

Adipose tissue mass is regulated by two main mechanisms: hypertrophy, defined as an increase in adipocyte size, and hyperplasia, defined as an increase in adipocyte number [20]. Adipose progenitor cells play a central role in this process as they give rise to new adipocytes and contribute to adipose tissue expansion and remodeling throughout life. Adipocyte progenitor pools are largely established during the prenatal period, whereas adipocyte number increases after birth and during adolescence, two critical windows for the subsequent development of obesity. In contrast, during adulthood adipocyte number tends to remain relatively stable, and adipose tissue expansion occurs predominantly through adipocyte hypertrophy [20–22]. In this context, alterations affecting adipose progenitor cells early in life may be particularly relevant as these cells may retain molecular features associated with prior metabolic exposure and potentially influence adipocyte differentiation, lipid handling, and metabolic responses later in life [19, 23–25]. Understanding whether such alterations are already established in childhood is important to clarify the early biological basis of obesity and its long-term consequences.

Adipose progenitor cells represent a key cellular substrate for obesogenic memory [19]. These cells are responsible for adipose tissue expansion and remodeling throughout life and may retain intrinsic molecular signatures reflecting prior metabolic exposure [25]; pediatric obesity induces stable, cell-autonomous reprogramming of adipose progenitors, and such changes could persist independently of systemic metabolic conditions and influence adipocyte differentiation, lipid handling, and metabolic responses later in life [23, 24]. Understanding whether these alterations are already established in childhood is essential to clarify the early biological imprinting of obesity and its long-term consequences.

To address this question, experimental models capable of isolating intrinsic cellular properties from systemic influences are required. In vitro differentiation of adipose-derived mesenchymal stem cells (MSCs) provides a controlled platform to investigate adipocyte development and metabolic programming while preserving donor-specific characteristics. However, standardized pediatric models are scarce, and the intrinsic properties of adipose progenitors from children with obesity remain poorly characterized.

In the present study, we developed and validated an in vitro model of adipogenic differentiation based on mesenchymal stem cells isolated from pediatric subcutaneous adipose tissue of normal-weight and obese subjects. Using this model, we aimed to determine whether pediatric obesity is associated with stable, cell-autonomous alterations consistent with an obesogenic memory. We hypothesized that, despite preserved differentiation capacity, adipose-derived progenitors from children with obesity would display persistent transcriptional and metabolic reprogramming affecting lipid metabolism and metabolic flexibility.

## Materials and Methods

### Study Population, Clinical Characterization, and Adipose Tissue Collection

Pediatric subjects undergoing elective minor surgical procedures were enrolled in the study, and periumbilical subcutaneous adipose tissue (scAT) samples were obtained intraoperatively. A total of 45 subjects (25 males and 20 females), ranging from 2 to 18 years (mean age 10.97 ± 4.10), were enrolled in the study. Each participant underwent standardized anthropometry, according to established pediatric protocols [26]. Height and weight were recorded. Measurements were obtained with subjects barefoot and wearing light clothing. Body weight was measured to the nearest 0.1 kg and height to the nearest 1 mm using a wall-mounted stadiometer. Waist circumference was assessed with a non-elastic tape midway between the lowest rib and the iliac crest. Body mass index (BMI) was calculated as weight/height^2^ (kg/m^2^) and converted into BMI z-scores according to WHO child growth standards [27]. Based on BMI z-score, participants were classified as normal weight (NW, −2 ≤ BMI z-score <1), overweight (OW, BMI z-score 1≤ BMI z-score <2), and obesity (OB, BMI z-score ≥2). Pubertal development was evaluated during the clinical examination using Tanner staging [28, 29], and participants were classified as stage 1 (Tanner 1), stage 2 (Tanner 2–3), or stage 3 (Tanner 4–5). Table 1 reports the demographic and auxological characteristics of the subjects, stratified according to BMI category.

**Table 1.** Demographic and auxological characteristics of children stratified according to BMI category

Variable	Normal weight, *n* = 23	Overweight, *n* = 7	Obesity, *n* = 15	*p* value
Sex, *n* (%)				
Female	10 (43.5)	3 (42.9)	7 (46.7)	0.977
Male	13 (56.5)	4 (57.1)	8 (53.3)	​
Age (mean±SD)	9.7±4.7	12.1±2.9	12.5±2.9	0.086
Weight (mean±SD)	36.9±20.0	55.3±17.0	62.8±11.1	<0.001
Height (mean±SD)	1.40±0.29	1.52±0.15	1.51±0.12	0.239
BMI (mean±SD)	17.5±3.8	23.2±2.8	27.3±2.7	<0.001
BMI z-score (mean±SD)	−0.20±1.21	1.78±0.16	2.56±0.61	<0.001
Waist circumference (mean±SD)	62.3±8.5	84.0±4.4	80.0±5.7	<0.001
Waist circumference/height ratio (mean±SD)	0.45±0.08	0.56±0.02	0.53±0.04	<0.001
Pubertal stage, *n* (%)				
Stage 1 (Tanner 1)	12 (52.2)	3 (42.9)	6 (40.0)	0.708
Stage 2 (Tanner 2–3)	2 (8.7)	2 (28.6)	3 (20.0)	​
Stage 3 (Tanner 4–5)	9 (39.1)	2 (28.6)	6 (40.0)	​

BMI, body mass index; SD, standard deviation.

### Isolation and Culture of Human Adipose-Derived Mesenchymal Stem Cells

Subcutaneous adipose tissue biopsies were processed to obtain human mesenchymal stem cells (hMSCs) using a non-enzymatic explant culture method [30–32]. Tissue fragments were placed in culture dishes and maintained in Dulbecco’s Modified Eagle Medium (DMEM) low glucose supplemented with F12 medium (1:1), 10% fetal bovine serum, and antibiotics at 37°C, 5% CO_2_. Migrating adherent cells were expanded and used for subsequent experiments.

From the 45 biopsies, cells were obtained from 23 samples. Of them, 10 were NW males and 5 NW females; on the other hand, 6 were from OB male subjects and 2 from OB female subjects. Given the low number of OB female samples and to avoid the introduction of sex-related confounding factors, subsequent analyses were restricted to cells derived from male subjects only (9 male subjects; for further details, see below and online suppl. Table S1; for all online suppl. material, see https://doi.org/10.1159/ofa/adzag003). Furthermore, to ensure robustness and reproducibility, only samples that consistently met predefined quality and differentiation criteria were included in the analyses (9 male pediatric subjects). Considering this, all the experiments on differentiated adipocytes were performed on cells derived from 6 NW to 3 OB subjects, all males. For the assessment of the method, cells from 3 normal-weight individuals (NW1, NW2, NW3) were utilized. Subsequently, for the working hypothesis validation and investigation, 3 NW, different from the assessment method (NW4, NW5, NW6), and 3 subjects with obesity (OB1, OB2, and OB3) were used. A flow diagram summarizing the sample selection process is shown in Figure 1.

**Fig. 1.**
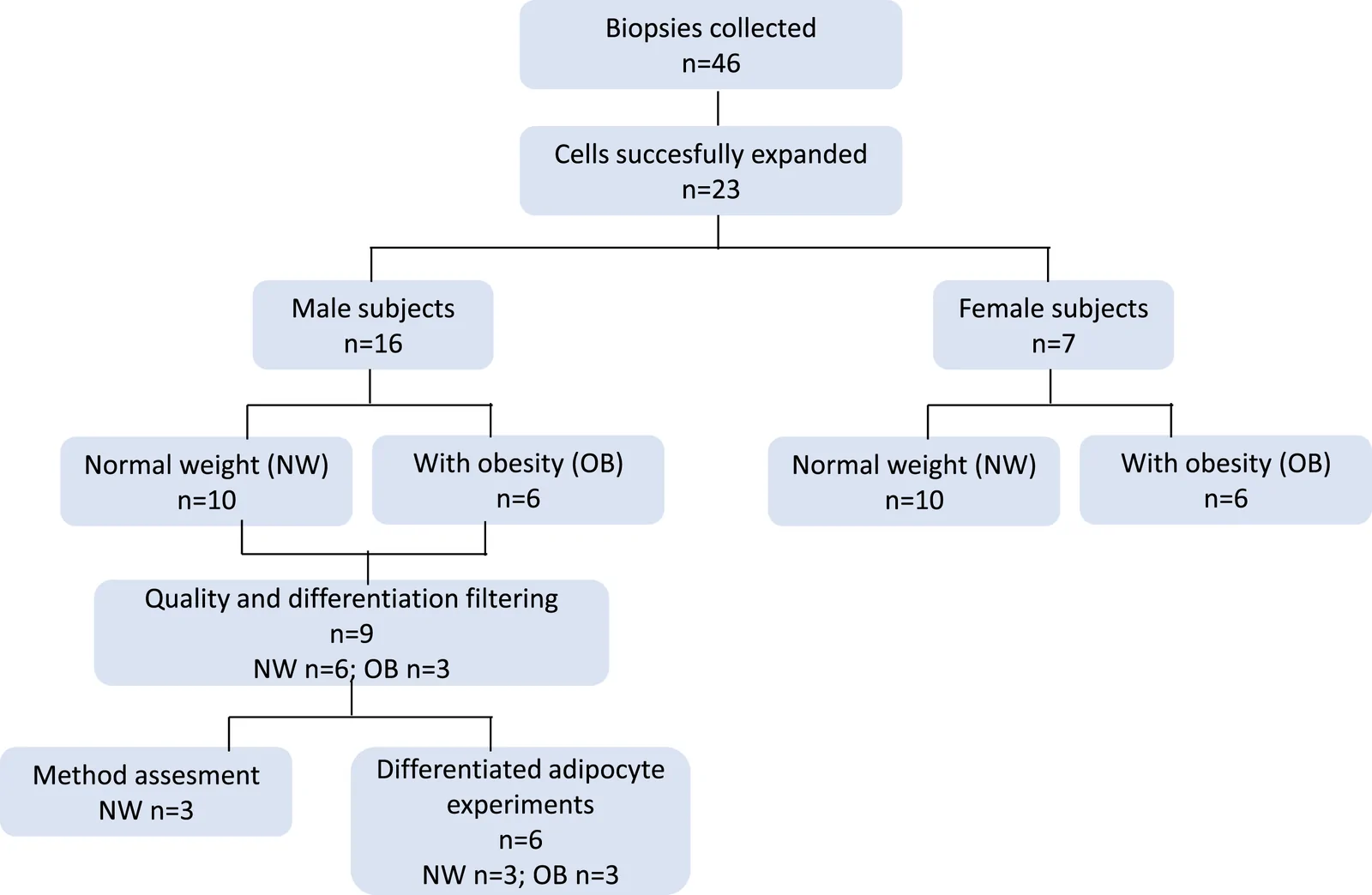
Flow diagram of human mesenchymal stem cells sample selection. Subcutaneous adipose tissue biopsies were collected from 45 pediatric subjects. Human mesenchymal stem cells (hMSCs) were successfully expanded from 23 biopsies, including samples from male with normal-weight (NW) and obese (OB) male and female subjects. To minimize sex-related confounding, only male-derived cells were selected for subsequent analyses. After quality and differentiation filtering, differentiated adipocyte experiments were performed on cells derived from 9 male subjects: 6 NW to 3 OB.

### In vitro Adipogenic Differentiation

hMSCs derived from normal-weight and overweight/obese subjects were expanded in culture and induced to undergo adipogenic differentiation using three adipogenic media (Protocols A, B, and C) (Table 2), differing in the concentration of pro-adipogenic factors including insulin, IBMX, dexamethasone, and indomethacin. The three differentiation protocols consist in a 14-day treatment evaluating the time points d0 (undifferentiated hMSCs), after 7 days (d7), after 10 (d10), and after 14 days of differentiation (d14). The main adipogenic factors added in the media included insulin, dexamethasone (DEX), which binds to the glucocorticoid receptor and activates the transcription of pro-adipogenic genes; 3-isobutyl-1-methylxanthine (IBMX), that is a non-selective phosphodiesterase inhibitor that triggers adipogenesis by increasing intracellular cAMP levels; and indomethacin, a PPARγ activator. All differentiation experiments were performed on cells deriving from 6 normal-weight subjects and 3 with obesity (Table 3).

**Table 2.** Composition of differentiation media A, B, and C

Differentiation medium A	Differentiation medium B	Differentiation medium C (induction medium)	Differentiation medium C (maintenance medium)
DMEM HG	DMEM HG	DMEM HG	DMEM HG
10% FBS	10% FBS	10% FBS	10% FBS
1% P/S	1% P/S	1% P/S	1% P/S
1% l-glutamine	1% l-glutamine	1% l-glutamine	1% l-glutamine
10 µL/mL insulin	10 µL/mL insulin	10 µL/mL insulin	10 µL/mL insulin
0.1 µm Dex	1 µm Dex	1 µm Dex	**//**
500 µm IBMX	500 µm IBMX	500 µm IBMX	**//**
200 µm indomethacin	200 µm indomethacin	//	**//**

**Table 3.** Primer sequences used for quantitative real-time PCR analysis

Gene	Forward primer sequence	Reverse primer sequence
*ACC*	GCT​GGC​TGG​GGT​CAT​GCT​TCT​G	GGC​TTG​GAG​GAC​CCA​TGA​AGG​C
*ACSL*	GGG​CGA​GGT​GTG​TGT​GAA​AGG​G	TCC​CCT​GTG​TGT​AAC​CAG​CCG​T
*ALPL*	GTG​TCT​CCA​CAG​TGA​CGG​CTG​C	TCT​TGG​AGA​GGG​CCA​CGA​AGG​G
*C/EBPA*	GGA​GCA​AAT​CGT​GCC​TTG​TC	CTT​CTC​TCA​TGG​GGG​TCT​GC
*CPT1A*	AGT​GCT​GCT​CTC​CTA​CCA​CGG​G	CGA​GGC​AGC​GAT​GTC​TGG​AAG​C
*FABP4*	CTG​GGC​CAG​GAA​TTT​GAC​GA	ACC​AGG​ACA​CCC​CCA​TCT​AA
*FASN*	AGG​CAC​ACA​CGA​TGG​ACC​CTC​A	GAC​GCC​AGT​GTG​TGT​TCC​TCG​G
*LIPE*	CTG​CTG​AGC​CTC​ATG​GAC​CCC​T	GGT​CCT​CCG​TCT​TTG​CAC​CAG​C
*MGLL*	CCT​TCT​GGG​CCA​CTC​CAT​GGG​A	GAG​TAC​CAT​GCC​GGC​GAA​GTG​G
*PNPLA2*	GCT​CAA​TGA​GGC​CCT​GCT​GGA​G	CGC​ACA​GGC​AGC​ATG​TTG​GAG​A
*PPARG*	TGC​CAC​AGG​CCG​AGA​AGG​AGA​A	AAT​GTT​TTG​CCA​GGG​ACC​CGG​AG
*U6*	GTG​CTC​GCT​TCG​GCA​GCA​AAT​A	CAT​CCT​TGT​GCA​GGG​ACC​ACG​C

### RNA Expression Analysis

For gene expression analysis, hMSCs 20.000 cells/well were plated into 12-well plates. The differentiation treatment started after 72 h of expansion and samples were taken at the established time points d0, d7, d10 and d14. Total RNA was extracted by organic extraction using TRIzol reagent (Invitrogen, Carlsbad, CA, USA). Total RNA concentration was measured using an Eppendorf BioSpectrometer. Complementary DNA (cDNA) synthesis was performed by reverse transcription, a total of 1,000 ng of RNA was used for each reverse transcription reaction. Reverse transcription was carried out in a final reaction volume of 10 µL, consisting of the RNA sample, 2 µL of reverse transcriptase (RR036A, Takara), and ultrapure water adjusted to the final volume. Reverse transcription reactions were performed using a Bio-Rad T100 Thermal Cycler according to the manufacturer’s protocol. The thermal program included three sequential steps: priming at 25°C, reverse transcription at 46°C, and enzyme inactivation at 95°C, for a total reaction time of 17 min. Following reverse transcription, each sample was diluted to obtain a final volume of 100 µL and a cDNA concentration of 10 ng/µL.

### Quantitative Real-Time PCR

Gene expression analysis was performed using TB Green Premix Ex Taq (Takara Bio) in combination with TaqMan chemistry to detect target genes. The reaction mixture for each well (final volume 10 µL) contained 5 µL TB Green master mix, 0.3 µL forward primer (10 µm), 0.3 µL reverse primer (10 µm) (Table 4), and 2.4 µL ultrapure water. Eight microliters of the prepared mix were dispensed into each well of a 96-well plate, followed by the addition of 2 µL cDNA sample. Amplification was performed using the CFX96 Connect Real-Time PCR Detection System (Bio-Rad Laboratories) according to the manufacturer’s recommended cycling program.

**Table 4.** Antibodies used for Western blot analysis

Target protein	Code	Dilution	Host
C/EBPα	CST, 2295S	1:1,000	Rabbit
CD36	Invitrogen, PA116813	1:1,000	Rabbit
CPT1A	CST, 97361S	1:1,000	Rabbit
FABP4	CST, 3544S	1:500	Rabbit
FADS1	CST, 10076S	1:1,000	Rabbit
GAPDH	Invitrogen, MA1-16757	1:2,000	Rabbit
PNPLA2 (ATGL)	CST, 2138S	1:1,000	Rabbit
PPARγ	CST, 2435S	1:1,000	Rabbit
SCD1	CST, 2794S	1:1,000	Rabbit
SREBP1	CST, 95879S	1:1,000	Rabbit
Vinculin	Abcam, 129002	1:1,000	Rabbit
β-actin	Sigma-Aldrich, A5441	1:1,000	Mouse

### Protein Extraction and Quantification

Total cellular proteins were extracted from in vitro differentiated adipocytes and analyzed by Western blot. hMSCs were seeded at 50,000 cells per well in 6-well plates. Adipogenic differentiation was initiated after 72 h of expansion, and samples were collected on days 0, 7, 10, and 14.

Proteins were extracted using radioimmunoprecipitation assay (RIPA) buffer (50 mm Tris-HCl pH 7.4, 150 mm NaCl, 1 mm EDTA, 1% NP-40, 1% sodium deoxycholate, 0.1% SDS) supplemented with protease inhibitor cocktail (Sigma-Aldrich). Cells were washed twice with PBS, lysed in RIPA buffer, and mechanically scraped on ice. Lysates were incubated on ice for 20 min with intermittent vortexing, then centrifuged at 15,000 rpm for 20 min at 4°C. Supernatants containing soluble proteins were collected.

Protein concentration was determined using the Bradford assay with Coomassie Brilliant Blue reagent. Samples or bovine serum albumin standards (Bio-Rad Laboratories) were added to Bradford reagent in 96-well plates, and absorbance was measured at 595 nm using a Thermo Fisher Scientific Multiskan spectrophotometer. Protein concentrations were calculated from a standard curve according to the Beer-Lambert law.

### Western Blot Analysis

Equal amounts of protein (20 µg) were mixed with Laemmli sample buffer containing β-mercaptoethanol and denatured at 95°C for 5 min. Proteins were separated by SDS-polyacrylamide gel electrophoresis. Proteins were transferred onto nitrocellulose membranes using a semi-dry transfer system (TransBlot Turbo, Bio-Rad Laboratories). Membranes were stained with Ponceau Red, washed in TBST, and blocked for 30 min in 5% bovine serum albumin with 0.05% Tween-20. Membranes were incubated for 18 h at 4°C with primary antibodies against adipogenic and metabolic markers (including C/EBPα, PPARγ, and FABP4) and loading controls (β-actin, vinculin) (Table 3). After washing, membranes were incubated with horseradish peroxidase-conjugated secondary antibodies for 90 min at room temperature. Immunoreactive bands were visualized by chemiluminescence using a UVITEC imaging system.

### Assessment of Adipogenic Differentiation and Lipid Accumulation

Adipogenic differentiation of hMSCs was evaluated by fluorescence-based lipid staining and morphological analysis. Intracellular neutral lipids were labeled using BODIPY 493/503 (10 µm Thermo Fisher Scientific), while cell nuclei were counterstained with Hoechst 33,342 (800 nm). Staining was performed at days 0, 7, 10, and 14 of differentiation.

Cells were washed with PBS and incubated for 20 min at 37°C with staining solution prepared in PBS (BODIPY 1:2,000; Hoechst 1:1,250). After incubation, cells were washed with PBS and maintained in PBS for imaging. Fluorescence images were acquired using a Logos Biosystems CELENA digital imaging system at ×40, ×100, and ×200 magnifications. Adipogenic differentiation was quantified by calculating the percentage of lipid-positive cells relative to total nuclei.

### Cell Viability Assessment (Live/Dead Assay)

To evaluate potential cytotoxic effects of adipogenic differentiation media, cell viability was assessed using a fluorescence-based Live/Dead assay. The assay distinguishes viable cells labeled with calcein-AM (green fluorescence) from non-viable cells labeled with ethidium homodimer-1 (red fluorescence).

hMSCs were seeded at 10,000 cells per well in 24-well plates and analyzed at days 0, 7, 10, and 14. After removal of culture medium and PBS washing, cells were incubated for 45 min at 37°C with staining solution prepared according to the manufacturer’s instructions (Live/Dead Assay Kit, Thermo Fisher Scientific) in α-MEM medium (Gibco). Cells were then washed with PBS and imaged in PBS. Fluorescence images were acquired at ×100 and ×200 magnifications.

### Statistical Analysis

Data are expressed as mean ± standard deviation (SD) of at least two independent experiments. Each experiment was performed as a biological duplicate that was analyzed independently in a technical duplicate. Statistical analyses were performed using GraphPad Prism. The normality of data distribution was assessed prior to analysis. For comparisons between two groups, statistical significance was evaluated using a two-tailed unpaired Student’s *t* test. For multiple-group comparisons, one-way analysis of variance (ANOVA) followed by appropriate post hoc tests was applied, as indicated. A *p* value <0.05 was considered statistically significant.

## Results

### Establishment and Validation of a Pediatric in vitro Model of Adipogenic Differentiation

To evaluate the efficiency of the adipogenic differentiation protocols, morphological changes and intracellular lipid accumulation were assessed throughout the differentiation period. Bright-field microscopy combined with lipid-specific staining revealed clear protocol-dependent differences in adipogenic induction. Cells treated with differentiation protocols A and B exhibited the progressive appearance and accumulation of intracellular lipid droplets, consistent with successful adipocyte maturation. In contrast, cells threated with protocol C maintained a fibroblast-like morphology throughout the experimental period and showed no detectable lipid droplet formation, indicating a failure to initiate adipogenic differentiation (Fig. 2a). Based on these findings, protocol C was excluded from all subsequent analyses.

**Fig. 2.**
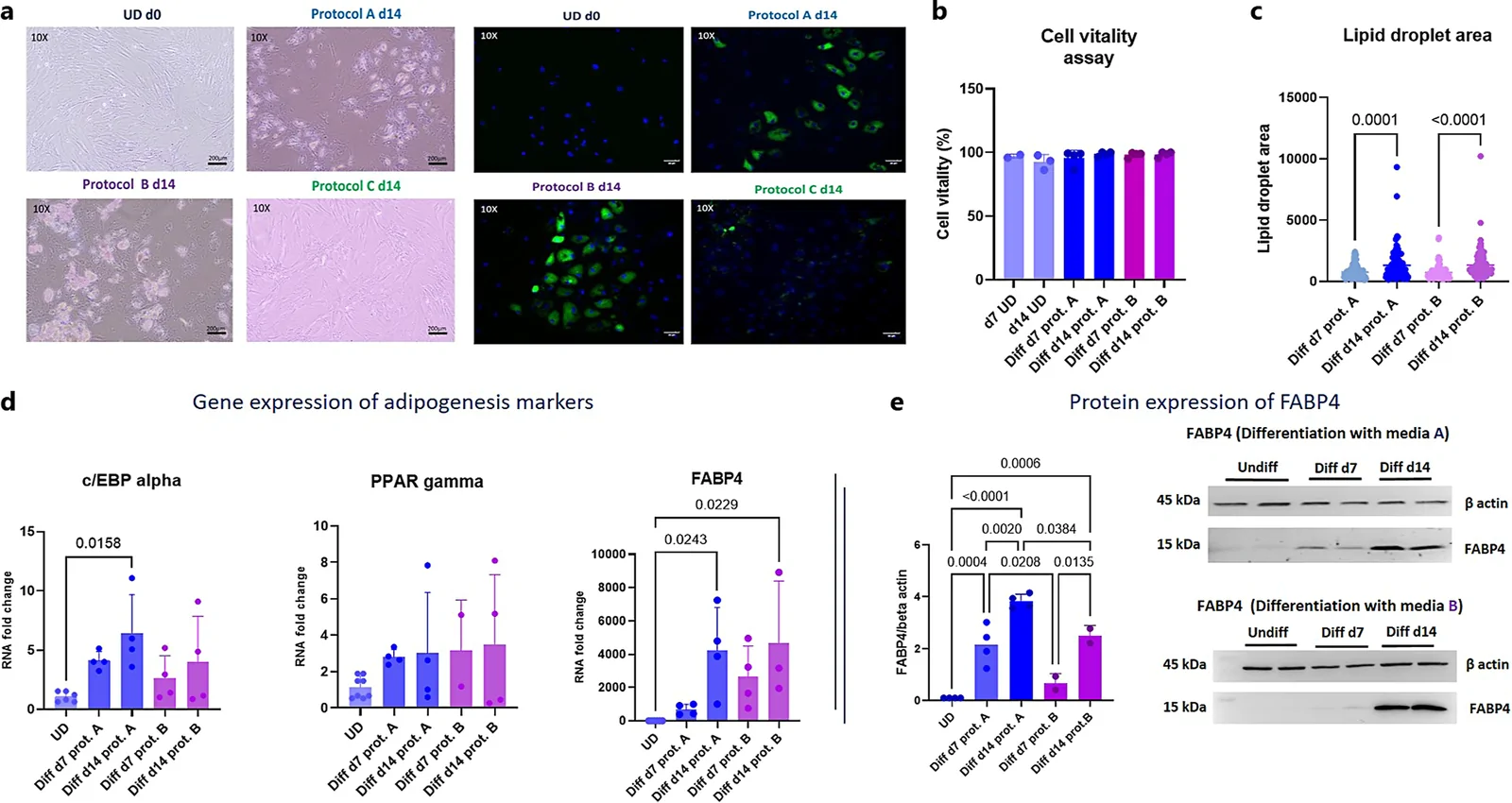
Pediatric adipocyte differentiation and evaluation of the effects of three different adipogenic media using hMSCs derived from normal-weight subjects (NW). **a** Bright-field and fluorescence images of undifferentiated and differentiated cells after 14 days of treatment with three differentiation media (A, B, and C). For fluorescence staining: nuclei in blue (Hoechst), lipid droplets in green (Bodipy). **b** Toxicity evaluation of the treatments after 7 and 14 days. **c** Lipid droplet size of cells after 7 and 14 days of adipogenic cocktails supplementation A and B. No further analysis was performed on protocol C because adipogenesis was not induced, resulting in complete absence of lipid droplets accumulation. **d** Molecular characterization of induced adipogenesis. mRNA expression of the transcription factors typical of adipogenesis (*C/EBPA*, *PPARG*, and *FABP4*) in undifferentiated cells, and cells at 7 and 14 days after supplementation of adipogenic cocktails A and B. **e** Protein expression of FABP4 during adipogenesis induced by adipogenic cocktail A and B after 7 and 14 days after supplementation. Data are presented as mean ± SD from cells derived from 3 different subjects (NW1, NW2, and NW3) performed in biological replicate. Each biological replicate was analyzed in technical duplicate, two statistical tools were used to verify the significance (*p* < 0.05), two-tailed unpaired Student’s *t* test and one-way ANOVA.

In order to exclude cytotoxic effects of the differentiation media, cell viability was assessed at both 7 and 14 days of treatment. Viability assays demonstrated that none of the differentiation conditions induced detectable cytotoxicity at either time point. Cell survival remained comparable across treatment groups and consistent with untreated controls (Fig. 2b).

Quantitative morphometric analysis further supported the morphological observations. Cells treated with protocols A and B exhibited a significant and time-dependent increase in lipid droplet size, reflecting progressive intracellular lipid accumulation and maturation of the adipogenic phenotype. Lipid droplets not only increased in number but also displayed enlargement over time, consistent with active lipid storage and functional differentiation. This temporal pattern was not observed in cells treated with protocol C, which showed no measurable changes in lipid content (Fig. 2c).

Furthermore, at the molecular level, the expression of mRNA of the adipocyte differentiation markers (*C/EBPA*, *PPARG*, and *FABP4*), was analyzed. Both *C/EBPA* and *PPARG* mRNA increased after 7 and 14 days of either media supplementation when compared to the undifferentiated condition. Also, no significant differences in marker expression were detected between cells cultured in media A and B after 7 and 14 days. A massive and significant increase in both *FABP4* mRNA expression has been observed at d7 and d14 of adipogenic cocktail treatment (Fig. 2d). The induction of FABP4 was further confirmed at the protein level, demonstrating strong concordance between transcriptional activation and protein accumulation (Fig. 2e).

### Comparison of Adipogenic Differentiation Capacity between Normal-Weight and Obesity-Derived Pediatric hMSCs

To determine whether pediatric obesity influences the intrinsic adipogenic potential of human pediatric mesenchymal stem cells (hMSCs), the differentiation efficiency of cells derived from normal-weight (NW) subjects and subjects with obesity (OB) following exposure to adipogenic protocol A. Morphological assessment performed after 14 days of differentiation revealed comparable cellular phenotypes between the two groups. As shown in Figure 3a, both NW- and OB-derived hMSCs underwent the characteristic morphological transition associated with adipocyte maturation, including cytoplasmic enlargement and prominent intracellular lipid droplet accumulation.

**Fig. 3.**
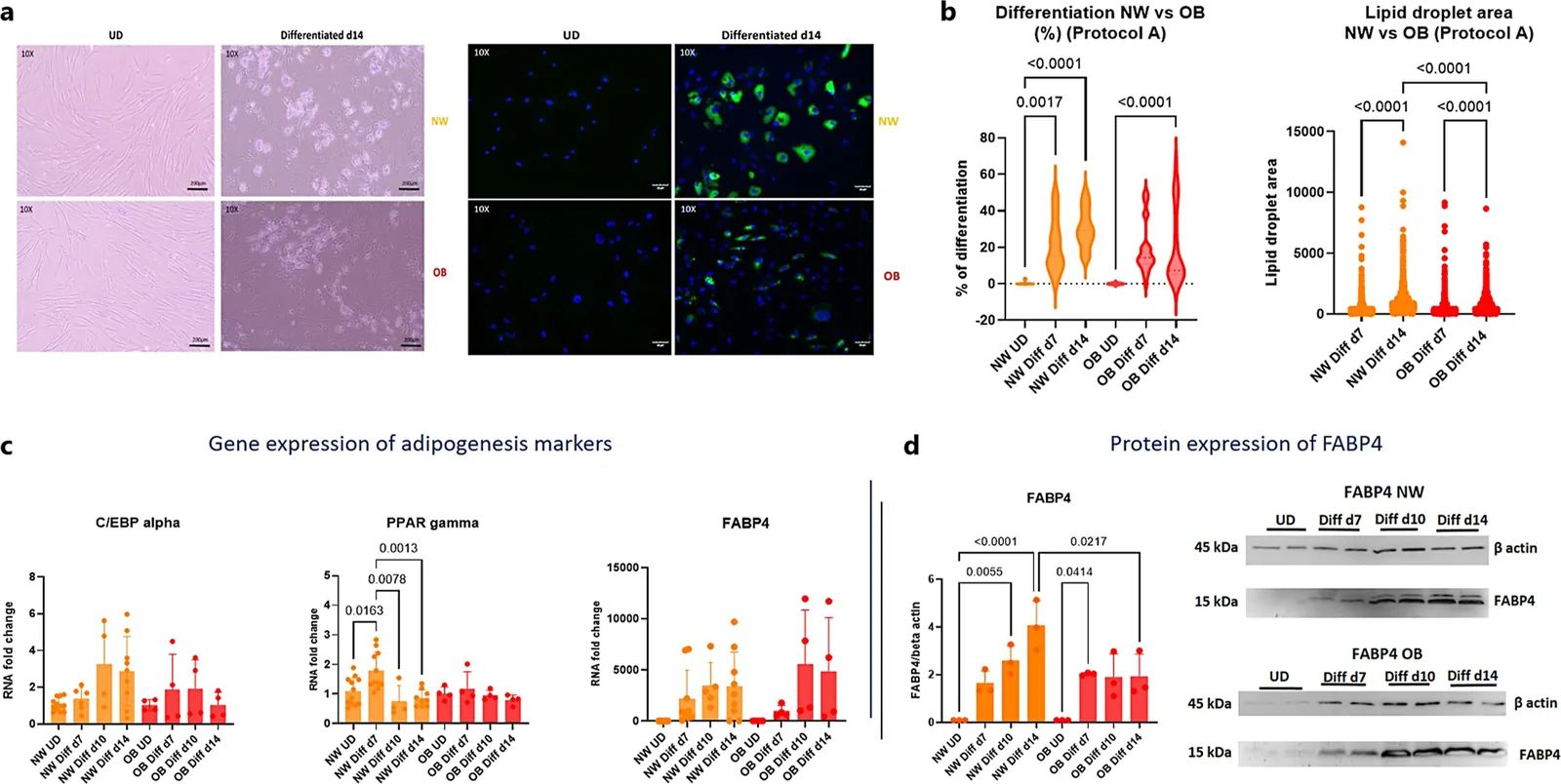
Adipogenic differentiation of hMSCs derived from NW and obesity (OB) cultured in adipogenic medium A. **a** Bright-field and fluorescence images of undifferentiated and differentiated NW- and OB-derived cells after 14 days of differentiation induction. For fluorescence staining: nuclei in blue (Hoechst), lipid droplets in green (Bodipy). **b** Quantification of cells undergoing differentiation (%) (left) and lipid droplet size of cells (right) induced by adipogenic cocktail A on cells derived from NW and OB and Lipid droplet size of cells derived from normal weight and with OB subjects undergoing differentiation induced by adipogenic cocktail A (on the right). **c** Molecular characterization of induced adipogenesis in hMSCs derived from NW and OB subjects. mRNA expression of the key adipogenic markers (*C/EBPA*, *PPARG*, and *FABP4*) in undifferentiated cells, and cells at 7, 10, and 14 days after supplementation of adipogenic cocktail A. **d** Protein expression of FABP4 during adipogenesis of hMSCs derived from NW and OB induced by adipogenic cocktail A. Data are presented as mean ± SD from cells derived from 3 different NW subjects (NW4, NW5, and NW6) to 3 different OB subjects (OB1, OB2, and OB3) performed in biological replicate. Each replicate was analyzed in technical duplicate, two statistical tools were used to verify the significance (*p* < 0.05), two-tailed unpaired Student’s *t* test, and one-way ANOVA.

To further quantify adipogenic outcomes, morphometric analyses were conducted to evaluate both the proportion of differentiated cells and lipid droplet size. These analyses demonstrated no statistically significant differences between NW- and OB-derived cultures in either parameter after 14 days of differentiation (Fig. 3b). The percentage of cells displaying lipid accumulation was comparable across groups, and the average size of lipid droplets, reflecting the extent of intracellular lipid storage, did not differ significantly. Molecular analyses further supported the morphological observations. No significant differences were observed in *C/EBPA* mRNA expression, although a trend of increased expression was noted in NW cells at days 10 and 14, whereas OB cells showed a flat, stable expression throughout differentiation. On the contrary, *PPARG* gene expression in NW- and OB-derived cells increased at day 7 and remained stable over the differentiation period. Expression levels of the maturation marker *FABP4* increased progressively over the course of differentiation, with comparable kinetics observed at days 7, 10, and 14 in both groups (Fig. 3c). This coordinated transcriptional program is consistent with normal adipogenic lineage commitment and maturation. Protein-level analysis confirmed these results. Western blot evaluation of FABP4 demonstrated similar levels of protein accumulation in differentiated adipocytes derived from NW and OB hMSCs (Fig. 3d), indicating that transcriptional induction was effectively translated into protein expression in both conditions.

### Obesity Is Associated with Transcriptional Reprogramming of Lipid Metabolism

Although no significant differences were observed in adipogenic efficiency between normal-weight (NW) and obesity-derived (OB) pediatric hMSCs, OB-derived cells were slightly less prone to undergo differentiation, and we hypothesized that obesity might be associated with qualitative alterations in adipocyte metabolic programming. Specifically, we investigated whether adipocytes derived from OB subjects exhibit distinct transcriptional regulation of metabolic pathways during differentiation, despite comparable morphological maturation.

To address this, we examined the expression of key genes involved in major lipid-metabolic processes, including lipogenesis, lipolysis, and fatty acid β-oxidation (Fig. 4). Genes involved in lipogenesis, including *FASN* and *ACC*, showed differentiation-dependent regulation in NW cells, reflecting activation of fatty acid synthesis during adipocyte maturation. Nevertheless, OB-derived adipocytes exhibited altered transcriptional dynamics, characterized by either reduced induction or irregular expression patterns compared with NW-derived cells.

**Fig. 4.**
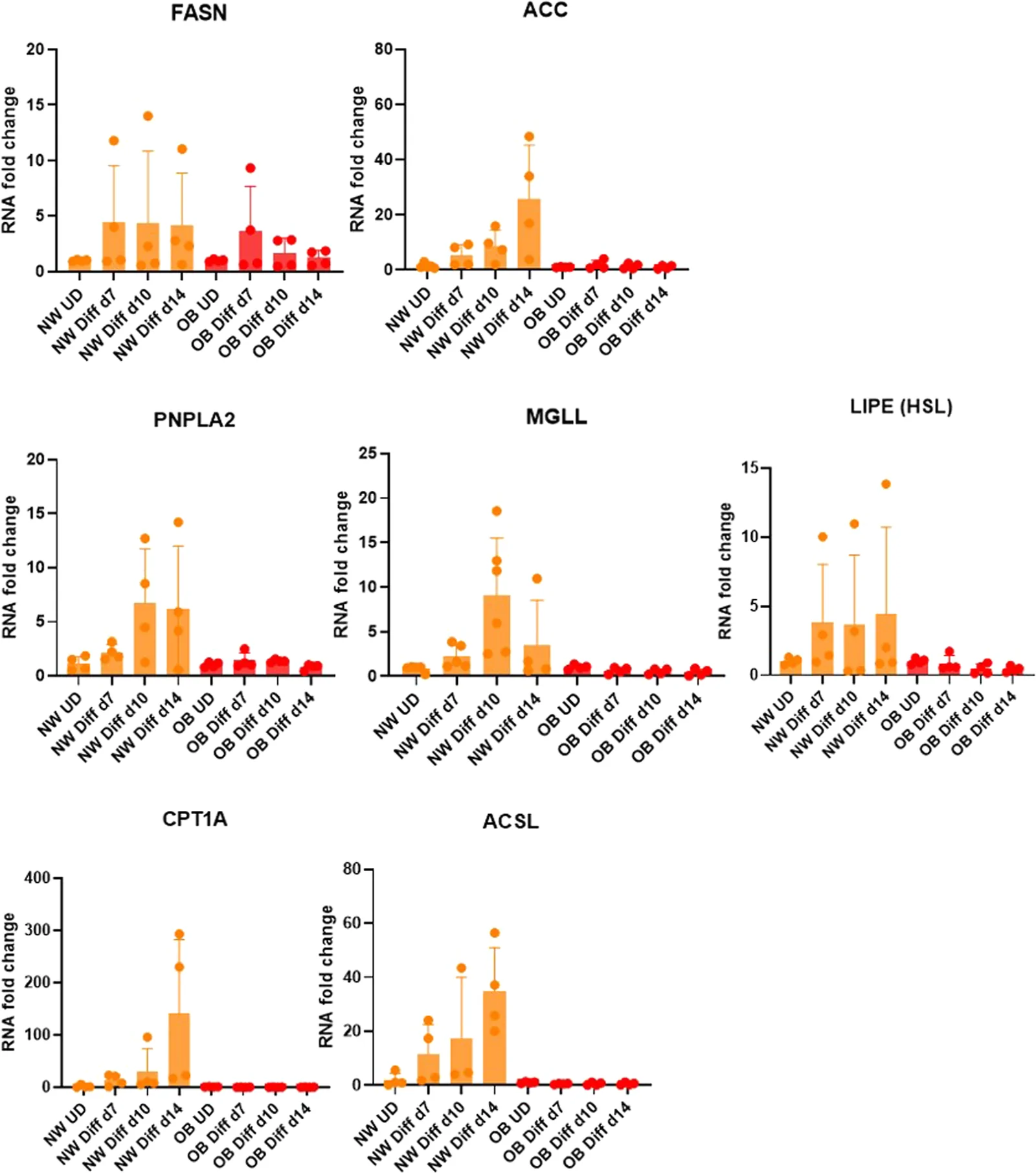
Insights about lipid metabolism in conditions of obesity and normal weight. Lipogenesis-related genes (*ACC, FASN*), lipolysis-related genes (*PNPLA2, LIPE, MGLL*), and β-oxidation-associated genes (*CPT1A, ACSL*) show increased expression in NW cells but remain stable in OB cells. Data are presented as mean ± SD from cells derived from 3 different NW subjects (NW4, NW5, and NW6) and 3 different OB subjects (OB1, OB2, and OB3). Each replicate was analyzed in technical duplicate. Two-tailed unpaired Student’s *t* test and one-way ANOVA were used to verify the significance (*p* < 0.05).

Similarly, the expression of key lipolytic genes *PNPLA2*, *MGLL*, and *LIPE*, which encode enzymes responsible for triglyceride mobilization, displayed divergent regulation between groups. While NW-derived adipocytes showed the expected differentiation-associated modulation of these genes, OB-derived cells demonstrated an attenuated or inconsistent transcriptional response, indicative of altered lipid turnover regulation.

Differences were also observed in genes involved in fatty acid β-oxidation, including *CPT1A* and *ACSL*. These genes play central roles in mitochondrial fatty acid transport and activation, respectively, and are critical for oxidative lipid metabolism. OB-derived adipocytes exhibited a blunted transcriptional adaptation in these pathways compared with NW-derived cells, suggesting reduced metabolic flexibility and potential impairment in lipid utilization.

This global divergence is further illustrated by the heatmap in Figure 5, which shows a clear separation not only between undifferentiated and differentiated cells but also between NW- and OB-derived adipocytes after 14 days of differentiation.

**Fig. 5.**
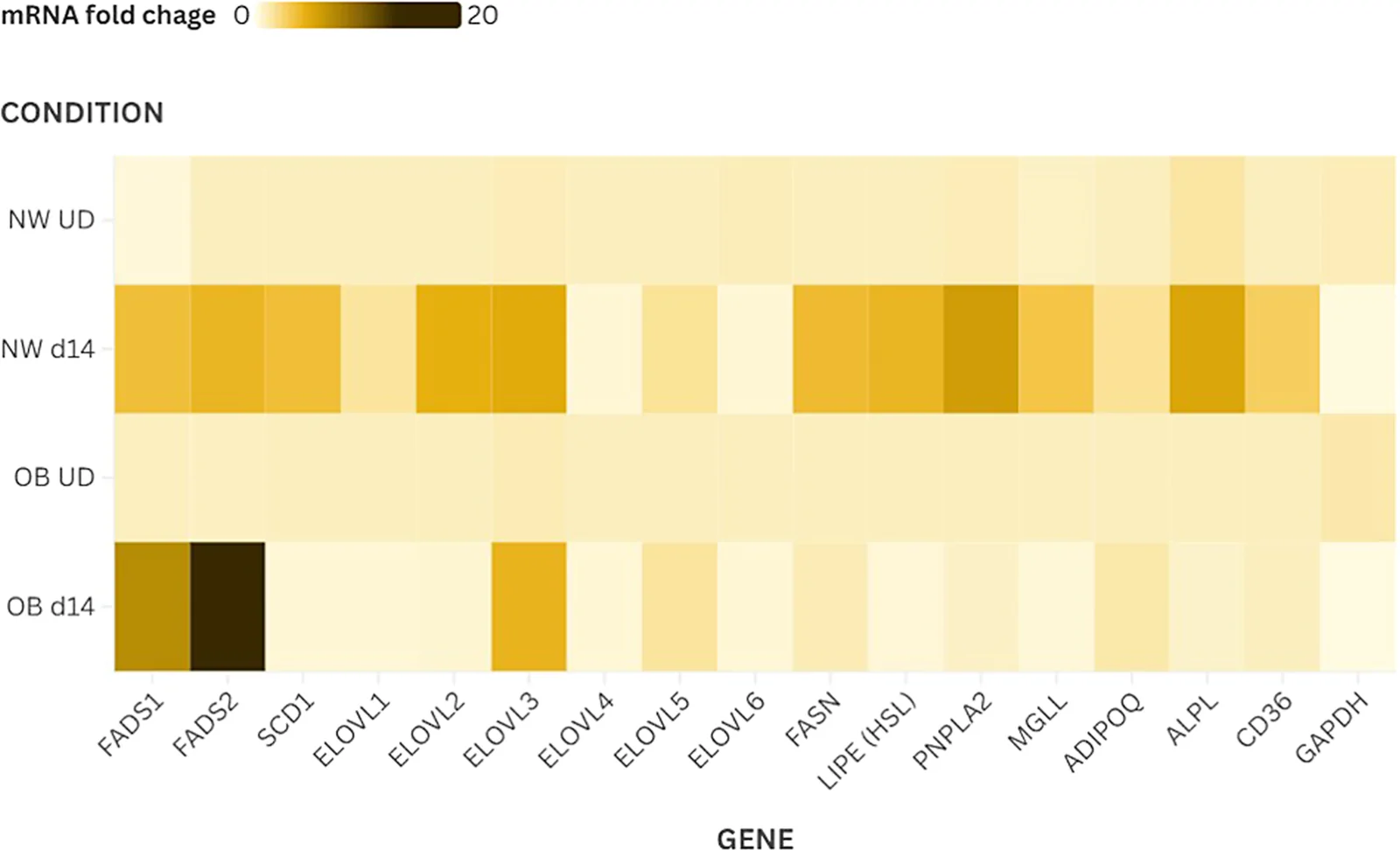
Expression levels of genes involved in lipid metabolism. Heatmap showing transcriptional differences of genes involved in lipid metabolism between undifferentiated hMSCs and differentiated adipocytes after 14 days of treatment with adipogenic media A derived from NW to OB subjects. Data are presented as mean ± SD from cells derived from 2 different NW subjects (NW4, NW6) and 2 different OB subjects (OB1, OB2) performed in biological replicate. Each biological duplicate was analyzed in technical duplicate, two statistical tools were used to verify the significance (*p* < 0.05), two-tailed unpaired Student’s *t* test and one-way ANOVA.

### Protein-Level Analyses Reveal Functional Metabolic Rewiring in OB-Derived Adipocytes

To determine whether the transcriptional differences observed between NW and OB adipocytes were accompanied by functional alterations at the protein level, we performed Western blot analyses of key regulators involved in lipid metabolism across adipogenic differentiation. This approach allowed us to assess whether the distinct transcriptional profiles identified in OB-derived cells translated into altered regulation of metabolic pathways governing lipid synthesis, mobilization, and remodeling.

Analysis of stearoyl-CoA desaturase 1 (SCD1), a central enzyme involved in monounsaturated fatty acid synthesis and lipid storage, revealed markedly different regulatory dynamics between groups. In NW-derived cells, SCD1 protein levels progressively increased during adipogenic differentiation. In contrast, OB-derived adipocytes displayed persistently elevated SCD1 expression that remained relatively constant across differentiation time points (Fig. 6a).

**Fig. 6.**
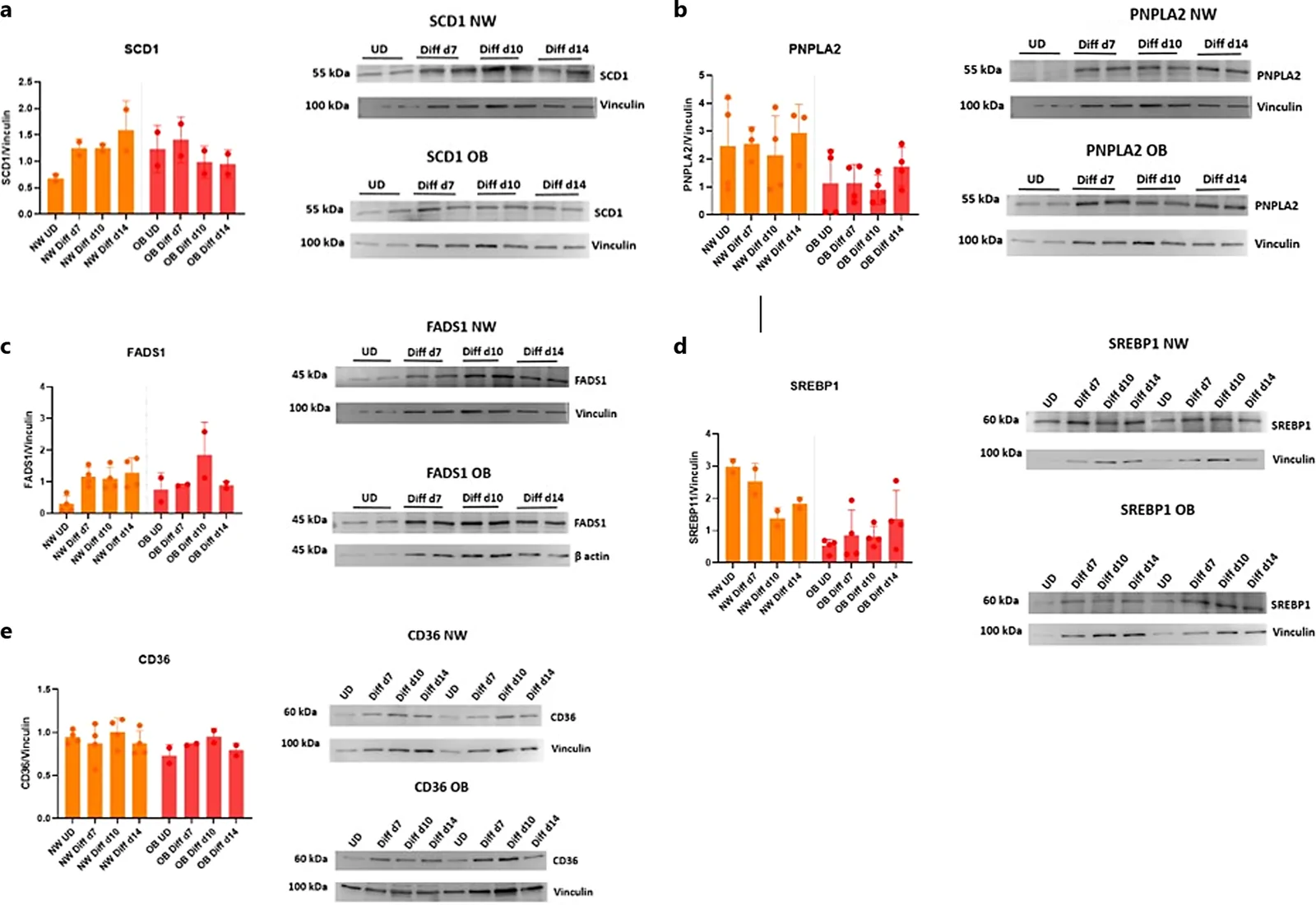
Protein expression of key lipid metabolism markers during adipogenesis in hADSCs derived from normal weight (NW) and with obesity (OB) subjects induced by adipogenic cocktail A after 7, 10 and 14 days of supplementation. **a** Protein expression of SCD1. **b** Protein expression of PNPLA2. **c** Protein expression of FADS1. **d** Protein expression of SREBP. **e** Protein expression of CD36. Data are presented as mean ± SD from cells derived from 2 different NW subjects (NW4, NW6) to 2 different OB subjects (OB1, OB2) performed in biological replicate. Each replicate was analyzed in technical duplicate, two statistical tools were used to verify the significance (*p* < 0.05), two-tailed unpaired Student’s *t* test, and one-way ANOVA.

We next examined patatin-like phospholipase domain-containing protein 2 (PNPLA2), a key enzyme mediating triglyceride hydrolysis and lipid mobilization. While PNPLA2 expression remained relatively stable throughout differentiation in both groups, OB-derived adipocytes consistently exhibited lower protein levels compared with NW-derived cells (Fig. 6b).

To further investigate lipid remodeling processes, we analyzed fatty acid desaturase 1 (FADS1), an enzyme involved in the synthesis of polyunsaturated fatty acids and the regulation of lipid composition. In NW-derived adipocytes, FADS1 protein levels increased progressively during differentiation, consistent with the acquisition of mature adipocyte lipid remodeling capacity. In contrast, OB-derived adipocytes displayed a markedly attenuated response, with minimal variation in protein levels across time points (Fig. 6c).

We also evaluated sterol regulatory element-binding protein 1 (SREBP1), a master transcriptional regulator of de novo lipogenesis that coordinates the expression of multiple genes involved in fatty acid and triglyceride synthesis. OB-derived adipocytes exhibited clear dysregulation of SREBP1 protein expression throughout differentiation compared with NW-derived cells (Fig. 6d).

In contrast to the marked differences observed in lipogenic and lipolytic regulators, the fatty acid transporter CD36 showed no significant differences in protein expression between NW- and OB-derived adipocytes at any differentiation stage (Fig. 6e). This finding indicates that cellular fatty acid uptake capacity is largely preserved in this model and suggests that the primary metabolic alterations associated with obesity occur downstream of lipid uptake, at the level of lipid processing, storage, and remodeling.

## Discussion

Childhood obesity represents an increasingly urgent global health challenge, with significant socioeconomic consequences if left unaddressed. Importantly, obesity established during childhood is strongly associated with the development of cardiovascular and metabolic complications later in life [33]. Pediatric obesity also tends to persist once established, suggesting that early metabolic alterations may become biologically embedded during childhood [34, 35].

The molecular mechanisms underlying this persistence remain largely unclear and have only begun to be investigated in recent years. Part of this knowledge gap stems from the complexity of adipose tissue biology and from the limitations of current experimental models used to study adipocyte development and function. A major challenge in adipogenesis research is the lack of standardized differentiation protocols, an issue that becomes even more critical when using primary cells derived from different donors, where inter-individual variability may substantially affect adipogenic potential. This challenge is particularly pronounced in pediatric models as adipogenesis in primary pediatric cells remains largely unexplored in the current literature. In addition, although childhood obesity is commonly identified using BMI in routine clinical practice because of the practical difficulties of directly assessing body composition, BMI does not fully capture adiposity. Accordingly, some children and adolescents who do not meet BMI criteria for obesity may still have excess body fat and an increased cardiometabolic risk [36]. This limitation should be considered when interpreting our findings as BMI-based classification may not fully reflect the biological and metabolic heterogeneity of pediatric adiposity.

In our study, for the first time, a reliable and efficient method to induce adipogenic differentiation in hMSCs derived from pediatric pWAT from NW and OB subjects was obtained. Among the adipogenic markers evaluated, FABP4 emerged as the most consistent and sensitive molecular indicator of successful differentiation. Therefore, our in vitro adipogenic differentiation model represents a valuable platform for investigating the molecular mechanisms governing adipogenesis and lipid metabolism in the context of pediatric obesity. Our results demonstrate that pediatric obesity is not merely characterized by an expansion of fat mass but is instead associated with a profound and persistent reprogramming of adipose progenitor cells that affects lipid-metabolic pathways and cellular metabolic flexibility.

This concept is in line with an increasing body of evidence indicating that adipose tissue dysfunction, rather than adipose tissue mass per se, is a major determinant of metabolic complications in obesity [11–13, 37, 38]. Several studies in adults have shown that obesity is associated with altered adipose tissue expandability, mitochondrial dysfunction, extracellular matrix remodeling, and chronic low-grade inflammation [39, 40]. However, much less is known about whether these alterations are already present and biologically imprinted during childhood. Our data provide direct experimental evidence that such reprogramming is already detectable at pediatric age and is maintained in isolated progenitor cells.

In addition, comparative analyses between NW- and OB-derived cells demonstrated that both groups retained the capacity to differentiate. This is consistent with previous reports indicating that obesity does not necessarily impair adipocyte commitment but rather affects the functional properties of differentiated adipocytes [11, 41, 42]. On the other hand, NW-derived adipocytes showed a significantly greater propensity for lipid droplet accumulation than OB-derived adipocytes. In addition, despite this apparently normal differentiation process, OB-derived adipocytes display a markedly altered metabolic phenotype, characterized by dysregulation of key pathways involved in lipid synthesis, lipid remodeling, and lipid mobilization. RT-qPCR analyses revealed unaltered expression patterns of key genes involved in lipid metabolism, including pathways related to lipogenesis, lipolysis, and fatty acid β-oxidation, in OB-derived differentiated cells compared with both NW-derived differentiated cells and undifferentiated controls. These results suggest the presence of obesity-associated alterations in adipocyte metabolic programming, supporting the concept that obesity may be linked to an intrinsic molecular reprogramming of adipose progenitor cells, especially in term, of gene expression related to lipid metabolism.

Furthermore, at the protein expression level, dysregulation of SCD1 and SREBP1 observed in our study is in line with previous work, where it has been shown that these factors play a central role in driving lipogenic programs [43, 44]. Indeed, increased SCD1 activity has been linked to enhanced triglyceride synthesis, altered membrane composition, and metabolic inflexibility, and its upregulation has been associated with insulin resistance and hepatic steatosis [45–47]. This lack of temporal modulation suggests dysregulated control of lipogenic signaling and indicates an intrinsic bias toward sustained lipid synthesis in OB-derived adipocytes, potentially reflecting a pre-existing pro-lipogenic metabolic state. Similarly, SREBP1 is a master regulator of de novo lipogenesis whose chronic activation contributes to ectopic lipid accumulation and metabolic disease [43, 48, 49]. This altered pattern suggests disrupted control of anabolic metabolic pathways and reinforces the notion that lipid biosynthetic signaling is fundamentally reprogrammed in adipocytes derived from obese pediatric subjects.

Conversely, the reduced expression of PNPLA2/ATGL observed in OB-derived adipocytes points toward impaired lipolytic capacity. Defective lipolysis has been previously described in obesity and is thought to contribute to adipocyte hypertrophy, lipid trapping, and impaired metabolic flexibility [50–52]. This reduction suggests an impaired lipolytic capacity in OB-derived adipocytes, which may contribute to altered lipid turnover and reduced metabolic flexibility. The combination of elevated lipogenic signaling and reduced lipolytic enzyme abundance supports the presence of a metabolic imbalance favoring lipid accumulation. In parallel, the blunted induction of FADS1 in OB cells suggests that obesity affects not only the quantity but also the quality of lipid species, in line with reports showing altered polyunsaturated fatty acid metabolism in obese adipose tissue. This blunted regulation indicates that obesity affects not only the quantity of lipid storage but also the qualitative aspects of lipid metabolism, including fatty acid composition and membrane remodeling processes.

This observation strongly supports the emerging concept of an epigenetically and metabolically imprinted “obesogenic memory,” whereby prior exposure to an obesogenic environment leaves stable, cell-autonomous marks in adipose progenitor cells that shape their differentiation trajectory and functional output. In this framework, obesity is not merely associated with reversible metabolic adaptations but with durable reprogramming of regulatory networks controlling lipid metabolism and cellular metabolic flexibility [18, 53]. Our data support this concept also in pediatric setting, suggesting that early-life obesity may leave a long-lasting biological imprint on adipose progenitor cells.

Several limitations of the present study should be acknowledged, including the relatively limited sample size, which reflects the intrinsic difficulty of obtaining pediatric adipose tissue samples. Furthermore, as commonly observed when working with primary human cells, inter-sample variability in both cell viability and differentiation potential was detected [54]. This variability reflects the intrinsic biological heterogeneity of primary cells. To ensure robustness and reproducibility, only samples that consistently met predefined quality and differentiation criteria were included in the analyses. In addition, the focus was restricted to cells derived from male subjects in order to avoid introducing sex-related confounding factors. In addition, while the in vitro model allows the identification of cell-autonomous alterations, it cannot fully recapitulate the complex multicellular interactions, immune cell crosstalk, and endocrine influences that characterize adipose tissue in vivo. Moreover, the cross-sectional nature of the study also prevents any direct inference on the temporal dynamics or causal relationships underlying the observed reprogramming.

Despite these limitations, our study provides evidence that pediatric obesity is associated with stable transcriptional alterations in adipose progenitor cell-derived adipocytes that persist under our experimental conditions. Our findings are compatible with the presence of an obesity-associated cellular memory, although this remains to be demonstrated by direct epigenetic and functional studies. While additional studies are required to further characterize the molecular and functional properties of these differentiated adipocytes, a deeper investigation of the proteomic and epigenetic differences observed in this model may contribute to a better understanding of the molecular mechanisms underlying obesity-associated metabolic alterations. Moreover, further investigations are needed to elucidate the molecular metabolic impact of obesity in females, thereby improving the understanding of sex-specific effects on adipocytes.

In conclusion, our findings support the view that pediatric obesity may be associated not only with increased fat mass but also with qualitative changes in adipose tissue biology. While the functional consequences of these changes remain to be established, these alterations may have implications for metabolic health and could help inform future studies aimed at identifying early preventive and therapeutic strategies.

## Statement of Ethics

The study protocol was reviewed and approved by the Ethics Committee Milano Area 1 (Milan, Italy; Approval No. 12/2016/20201). Informed written consent was obtained from the parents and/or legal representatives of the patients after they had received information about the study. The study was conducted in accordance with the Declaration of Helsinki.

## Conflict of Interest Statement

The authors declare that they have no conflict of interests.

## Funding Sources

The project received contributions from PRIN: progetti di ricerca di rilevante interesse nazionale-bando 2022 prot. 20228pnnjl.

## Author Contributions

Conceptualization, G.P., S.C., G.Z., and V.C.; methodology, M.D., C.B., G.P., M.B., F.D., G.C., M.C., A.L., S.C., G.Z., and V.C.; investigation, M.D., C.B., G.P., M.B., F.D., G.C., M.C., and A.L.; formal analysis M.D., C.B., M.B., and S.C.; writing – original draft preparation, M.D., C.B., M.B., F.D., G.C., A.L., S.C., and V.C.; writing – review and editing, M.D., C.B., G.P., A.L., S.C., G.Z., and V.C.; supervision, S.C., G.Z., and V.C; and funding acquisition, G.P., G.Z., and V.C. All authors have read and agreed to the published version of the manuscript.

## Data Availability

All data generated or analyzed during this study are included in this article and its supplementary material files. Further inquiries can be directed to the corresponding author.
